# Deformation Effects in Magnetic Guides: Challenges and Solutions with Sensors

**DOI:** 10.3390/s25226838

**Published:** 2025-11-08

**Authors:** Berend Denkena, Henning Buhl, Jingcai Zhang

**Affiliations:** 1 Institute of Production Engineering and Machine Tools, Gottfried Wilhelm Leibniz University Hannover, 30823 Garbsen, Germany; denkena@ifw.uni-hannover.de (B.D.); buhl@ifw.uni-hannover.de (H.B.); 2 Cluster of Excellence PhoenixD, Gottfried Wilhelm Leibniz University Hannover, 30167 Hannover, Germany

**Keywords:** deformation, guideways, production equipment, magnetic materials, optic measurement

## Abstract

Magnetic guides facilitate frictionless movement in machine tools, allowing for high position accuracy and high feed rate dynamics. However, achieving a stable state while subjected to machining forces requires a high magnetic force, which will cause deformation of the surrounding construction. This deformation leads to the contraction of the air gap between the slide and guideways. The air gap has a quadratic effect on the magnetic force, which poses a challenge to the control system. In addition, the effect of elastic deformation on magnetic guides is difficult to detect in real time due to the interference of magnetic fields in sensors and installation space constraints. In order to quantitatively analyze the effects of magnetic guide deformations, particularly of electrical steels, a combined approach of numerical analysis and experimental evaluation was conducted. As a result, the deformation of electrical steels, which is typically overlooked in conventional applications, is identified and analyzed. Force stabilization is achieved through adaptive set point correction and current adjustment, ensuring reliable and accurate operation of the magnetic guide system.

## 1. Introduction

Milling or grinding processes require high positioning accuracy while withstanding high process forces. But the friction and finite stiffness of guides limit the positioning accuracy. Compared to conventional contact-based guides, such as friction guides or rolling guides, magnetic guides are primarily used in precision positioning applications [1,2]. They offer frictionless movement, high damping and a theoretically infinite stiffness at sufficiently high forces [3,4,5]. Moreover, active multi-dimensional position compensation is an advantage of magnetic guides [6,7]. In some applications of machining processes, they are typically paired with linear direct drives to eliminate mechanical contact between the moving slide and the fixed component, enabling precise machining operations [8,9,10] or positioning with multiple degrees of freedom [11,12,13,14]. When coupled with integrated sensor capabilities, such as position compensation and force reconstruction, these features significantly simplify the implementation of adaptive machining strategies [15,16].

As such systems require no lubricants and show no mechanical wear, they are practically maintenance-free. In general, there are two types of magnetic levitation systems: The first is based on the Lorentz force, and its open-loop systems often exhibit lower stiffness compared to contact-based platforms, such as planar transport machines [17,18,19]. The second type utilizes magnetic reluctance forces as reluctance actuators, which always have negative stiffness, offering high force densities while minimizing heat dissipation and reducing actuator mass [20,21]. This type is often used in machining or production areas [14,22,23]. In reluctance-based magnetic levitation platforms, the in-process positioning of the levitated workpiece is governed by the reluctance force generated between the actuating electromagnet and the ferromagnetic back-iron. This interaction yields a strongly nonlinear force-gap-current relationship, in which the net attraction increases with the coil current and decreases with the air-gap length. By deliberately varying the magnetic reluctance force through modulation of the coil current and, where applicable, adjustment of the effective magnetic path, precise position control (i.e., fine position adjustment) can be achieved [24,25].

However, there is a non-linear and complex relationship between the air gap and the input current of the magnet for controlling the reluctance force. The reluctance force is dependent on the current through a quadratic term. In addition, practical implementations must account for material saturation, eddy-current and thermal effects [26,27,28], and input and state constraints [29] such as allowable current, temperature rise, and minimum/maximum air gap. Within this framework, feedback or feedforward control laws regulate the reluctance force to track commanded positions while maintaining stability and robustness against process disturbances and parameter variations [30,31].

In addition, structural deformation is a crucial factor affecting the positioning accuracy of machine tools under machining forces [32,33]. Similar to conventional guide systems, deformation in magnetic guide systems affects the desired air gap between the magnet and the ferromagnetic material. Variations in the air gap result in corresponding changes to the magnetic flux density at the surface of the electromagnet, which in turn alters the reluctance force. These variations challenge the design of controllers in ensuring satisfactory performance, stability and disturbance robustness [34,35].

The deformation of the back-iron and magnetic core is often a challenge in those applications [36,37]. Lamination stacks, which are formed by multiple stacked steel sheets, exhibit highly complex mechanical responses: interlayer contact and micro-slip, anisotropy, preload and friction effects, local plasticity, and manufacturing and assembly tolerances. Temperature- and frequency-dependent behaviors also act jointly to produce pronounced nonlinearities, making their behavior fundamentally challenging to model [38,39]. Consequently, in machining applications that employ magnetic guides, it is essential to account for the deformation of the back-iron and magnetic core [40]. These components are typically fabricated from laminated electrical steel, yielding lower stiffness than conventional structural steels. While capacitive or inductive sensors can indirectly measure the coordinate position of a levitated slide, accurately quantifying the deformation of the magnetic components remains difficult due to strong magnetic fields, stringent precision requirements, and the restricted installation space [14,41,42]. Thus, deformation of the back-iron and magnetic core often becomes a critical bottleneck for both modeling fidelity and experimental characterization in such systems. As a result of the mechanical deformations and the associated nonlinearities, incorrect force estimation and possible collision to the guideways remain a challenge for the application of magnetic guides in machining.

This paper is organized as follows: First, the overall concept and principles of magnetic guides are introduced in Section 2. Further, the limitations of the deformation simulation approach are mentioned, and afterwards, the challenges of the conventional measurement system are discussed (Section 3). Next, the deformation of the support structure caused by an increase in reluctance force is demonstrated (Section 4), followed by the deformation measurement of the back-iron using an optical measurement system (Section 5). Finally, the effects of magnetic guide deformation with an increasing magnetic reluctance force are analyzed and possible solutions to this phenomenon are determined based on the analyzed configuration (Section 6).

## 2. Construction of Magnetic Guides

### 2.1. Principles of Magnetic Guides

**Figure 1:**
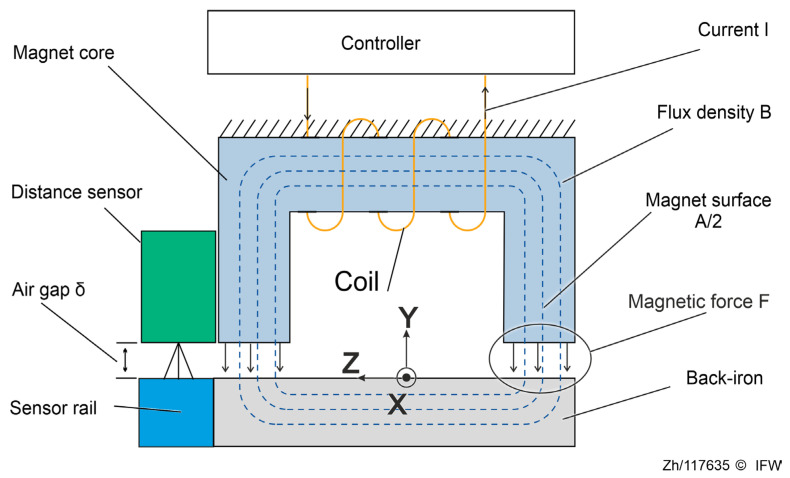
Working principle of an electromagnet.

Magnetic guides that are based on the reluctance force principle consist of electromagnets (magnetic actuators), which generate attracting forces against the back-iron of the guideway. Figure 1 shows the working principles of an electromagnet and illustrates the layout of the most relevant parts.

As the current flows through the coil around the magnet core, it generates a magnetic field. The magnetic flux passes through the magnet core, forming a closed loop through the air gap with the back-iron. This creates an attracting force *F* (reluctance force) between the core’s opening and the back-iron. Simultaneously, the distance sensor shown in the left corner of Figure 1 continuously detects the length of the air gap and transmits real-time feedback to the control system.

Based on the sensor’s feedback, the control system adjusts the current to precisely control the strength of the magnetic force, enabling accurate positioning and motion control. The magnetic force between a magnet and a back-iron can be expressed approximately using Maxwell’s formula for the iron-to-air transition tension. After a series of simplifications, it can be described as follows:

(1) $$F=\frac{B^{2}\cdot A}{2\mu_{0}}=\frac{N^{2}\cdot A\cdot \mu_{0}}{8}\cdot \frac{I^{2}}{\delta^{2}}$$

Here, *N* represents the number of turns in the coil, whereas *A* denotes the cross-sectional area of the flux path in the electromagnet. The vacuum permeability is represented by *μ*_0_ and *B* is the average value of the magnetic flux density. The force *F* represents the reluctance force, which is inversely proportional to the square of the air gap *δ* and directly proportional to the square of the current *I*. This approximate formula describes the fundamental behavior of the magnetic force between the magnet and the ferromagnetic material.

For convenience, the constant is defined as:

(2) $$C=\frac{\mu_{0}\cdot N^{2}\cdot A}{8}$$

And

(3) $$F=C\cdot \frac{i^{2}}{\delta^{2}}$$

assuming the operating equilibrium point is $\left(i_{0},\delta_{0}\right)$. When the system experiences small perturbations, the current and air gap can be expressed as follows:

(4) $$i=i_{0}+\Delta i\;\;\mathrm{and}\;\;\delta =\delta_{0}+\Delta \delta$$

A first-order Taylor expansion is performed on $F=\left(i,\;\delta \right)$ to linearize the small perturbations as follows:

(5) $$\Delta F\approx {\left.\frac{\partial F}{\partial i}\right|}_{\left(i_{0},\delta_{0}\right)}\Delta i+{\left.\frac{\partial F}{\partial \delta }\right|}_{\left(i_{0},\delta_{0}\right)}\Delta \delta$$

Based on Equation (3), the partial derivatives can be calculated as follows:

(6) $${\left.\frac{\partial F}{\partial i}\right|}_{\left(i_{0},\delta_{0}\right)}=2C\frac{i_{0}}{\delta_{0}^{2}}$$

(7) $$\;{\left.\frac{\partial F}{\partial \delta }\right|}_{\left(i_{0},\delta_{0}\right)}=-2C\frac{i_{0}^{2}}{\delta_{0}^{3}}$$

Therefore, the deviation of the approximate formula is expressed as:

(8) $$\Delta F=2C\frac{i_{0}}{\delta_{0}^{2}}\Delta i-2C\frac{i_{0}^{2}}{\delta_{0}^{3}}\Delta \delta$$

Moreover, in applications of magnetic guides, the stiffness also depends on the accurately measured instantaneous linearized position and its feedback controller, defining the feedback control as the transfer function G(s) and the air gap error as $\;e=\delta_{0}-\delta$. In the frequency domain, the differential equation for the closed loop is consequently:

(9) $$\Delta i=G\left(j\omega \right)\;\cdot \Delta e=-G\left(j\omega \right)\cdot \Delta \delta$$

Substituting into Equation (8) yields:

(10) $$\begin{array}{c} \Delta F=-2C\frac{i_{0}}{\delta_{0}^{2}}G\left(j\omega \right)\cdot \Delta \delta -2C\frac{i_{0}^{2}}{\delta_{0}^{3}}\Delta \delta ,\\ \;\;\Delta F=\left[-2C\frac{i_{0}^{2}}{\delta_{0}^{3}}-2C\frac{i_{0}}{\delta_{0}^{2}}G\left(j\omega \right)\right]\Delta \delta \end{array}$$

For convenience, the equation can be expressed with a positive sign as follows:

(11) $$\Delta F=K_{\mathrm{eff}}\left(j\omega \right)\cdot \Delta \delta$$

(12) $$K_{eff}\left(j\omega \right)=2C\frac{i_{0}^{2}}{\delta_{0}^{3}}+2C\frac{i_{0}}{\delta_{0}^{2}}G\left(j\omega \right)$$

$K_{eff}\left(j\omega \right)$ represents the dynamic stiffness coefficient of a single electromagnet with respect to air gap variations in the frequency domain.

In summary, the magnetic reluctance force is strongly quadratic with respect to the air gap δ. Thus, small gap errors are greatly amplified. The resulting dynamic stiffness comprises the sharp nonlinear static magnetic term and the closed-loop contribution introduced by the transfer function $G\left(j\omega \right)$. Consequently, any underestimation not only biases the force estimate, but also shifts the linearization operating point and loop gains, leading to distorted evaluations of $K_{eff}\left(j\omega \right)$ and reduced stability margins. Therefore, accurate calculation (and online maintenance) of the air gap is a prerequisite for reliable force estimation and dynamic-stiffness identification in magnetic guidance systems, and should be ensured through rigorous calibration, compensation, and consistency monitoring.

### 2.2. Magnetic Guides in Machining Tools

**Figure 2:**
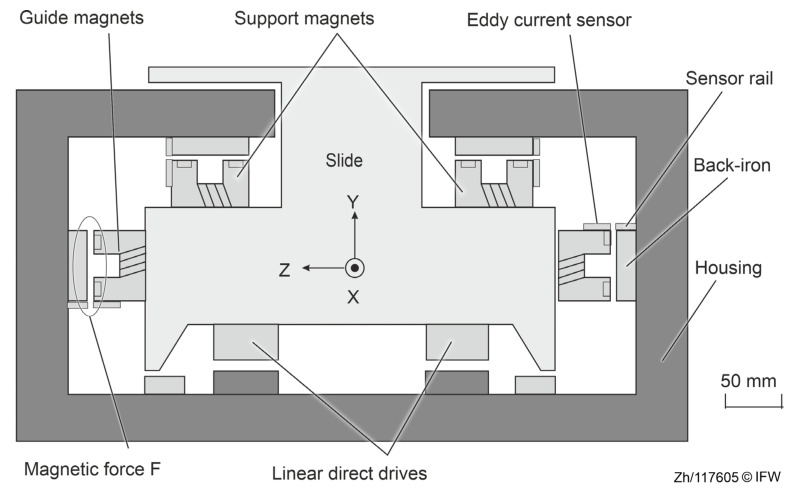
Active magnetic guides in a transport system.

The guide system in a machine tool significantly impacts its overall performance [2]. Magnetic guides control the position of a slide or machining axis by rapidly adjusting the reluctance force between the electromagnets and back-iron. A typical mutually perpendicular magnet configuration used in magnetic guides is shown in Figure 2. The support magnets and guide magnets stabilize the guide in the desired degrees of freedom. In addition, linear direct drives can also be used to achieve the feed movement of the slide or machining axis.

In many applications, the air gap in reluctance-force-based systems typically lies within the millimeter range. At such small scales, the nonlinear relationship between the reluctance force and the air gap becomes particularly significant, significantly complicating the control strategy and reducing overall system performance. Under high machining forces, even tiny changes in the air gap can have a substantial impact on the reluctance force generation, thereby affecting the stability and accuracy of the magnetic guide system.

## 3. Challenges

In the application of magnetic guides in machining, a number of issues resulting from the elastic deformation of the guideway’s construction must be considered due to the small distance between the slide and guideway. The reluctance force exerted on the guideway or magnet itself induces elastic deformation, which subsequently shifts the actual position of the operating point and influences the force estimation. Consequently, improper estimation of the set point and the force during operation affect the performance of the magnetic guide system. To ensure accurate force estimation and avoid possible collision with the guideways, it is essential to understand how structural deformation affects the reluctance force performance and what the results could be. Therefore, this paper aims to systematically investigate deformation behavior and its impact on magnetic guide performance under machining conditions.

**Figure 3:**
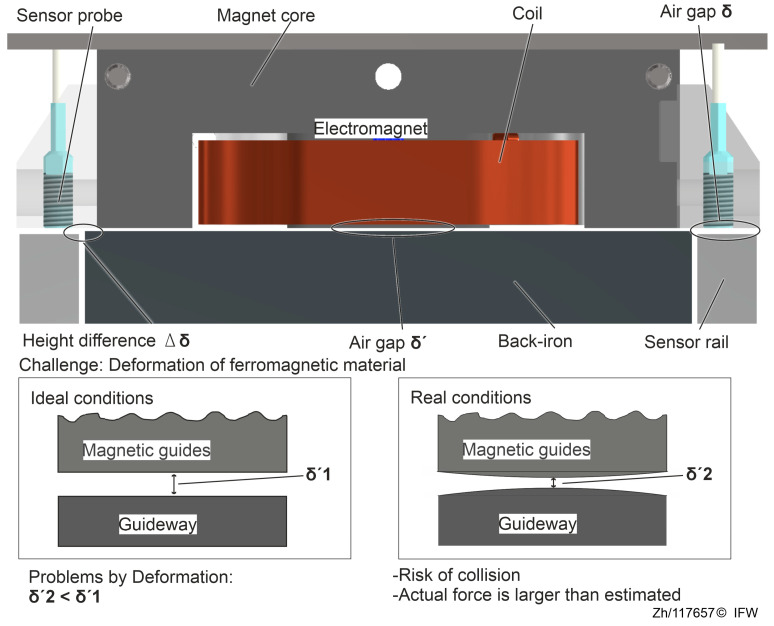
Deformation under the reluctance force.

First, a challenge description is provided for this deformation issue. The basic structure of a magnetic guide consisting of a magnet and a back-iron in one degree of freedom is shown in Figure 3. The plane of the back-iron and the plane of the sensor rail are parallel and have a height difference. The distance $\delta^{\prime }$ between the magnet and the back-iron is obtained indirectly by the high-speed eddy current sensor measuring the distance δ between the probe surface and the sensor rail plane.

Similar to synchronous electric motors, both the magnetic core and the back-iron are typically manufactured laminated. This manufacturing method effectively minimizes eddy current effects within the structure. This contributes to the enhanced dynamic response of the magnetic guides. However, the laminated components are less stiff than structural steel. Their deformation is affected by the inherent properties of the material, the processing method and the fastening method. In addition, the deformation cannot be considered as a rigid body, making it more difficult to determine realistic deformation results through a static-mechanical FE simulation.

**Figure 4:**
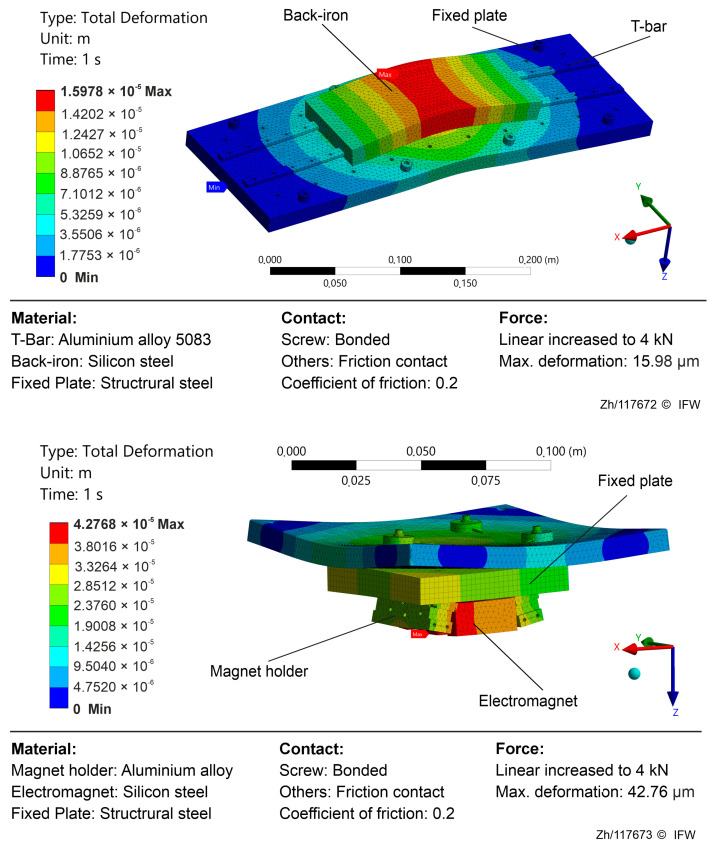
Deformation based on ANSYS 2025 R1.

As illustrated in Figure 4, simulations were performed for a single-degree-of-freedom model, treating the electromagnet and the magnetic guideway separately. The simulated fields indicate that the largest deformation is concentrated within the ferromagnetic region. The observed deformation trends point to three principal contributors: test rig compliance, magnetic-assembly compliance, and hysteresis deviation. Rig compliance denotes the global deformation of the test bench and mounting structure. Under a high magnetic reluctance force, elastic deflection of the base, fixtures, and sensing rail causes the indirect air gap δ measured by the eddy current sensor to deviate from the true core to back-iron gap $\delta^{\prime }$. Strong reluctance forces compress the laminations, back-iron, and their adhesive interfaces, reducing the gap in a manner not fully captured by the eddy-current measurement path. This mechanism grows nonlinearly with force, emerges as the dominant source of estimation bias in the high-load regime, and drives the operating point toward a smaller effective gap. In the low-magnetization regime, material hysteresis and eddy-current losses introduce path dependence in the force–flux relationship, while simplified formulations (e.g., reluctance force models) that neglect saturation and leakage flux yield additional systematic error.

Thus, the regular construction shown in Figure 3 accounts only for the influence of deformations in other parts and does not measure the elastic deformation of the back-iron and magnet core. Based on that assumption, it is assumed that the actual air gap $\delta_{2\;}^{\prime \;}$ between the magnet and the back-iron is smaller than the measured air gap δ  obtained by the eddy current sensors. In addition, eddy currents and magnetic flux generated in the magnet core and back-iron can interfere with the normal operation of inductive sensors. As a result, the effective management of potential deformations remains a key challenge when integrating a magnetic guide system into a production environment.

## 4. Analysis

As discussed in the previous sections, deformation is an unavoidable challenge for the application of reluctance force magnetic guides. Under high magnetic attraction, the magnetic guideway undergoes elastic deformation, causing the true air gap $\delta^{\prime }$ to deviate from the indirect air gap δ inferred by a conventional eddy current probe. At small gaps and high currents, this discrepancy is amplified by the nonlinear force–gap–current relationship. Under the Maxwell approximation in Section 2, the leading term of the magnetic attraction satisfies $F\propto \frac{I^{2}}{\delta^{2}}$ (neglecting saturation and higher-order corrections). Hence, the minimal-assumption, order-of-magnitude first-order sensitivities are

(13) $$\frac{\partial ln\;F}{\partial \ln I}\approx 2,\;\frac{\partial ln\;F}{\partial \ln\delta }\;\approx -2\;$$

So, small perturbations obey

(14) $$\frac{\Delta F}{F}\approx 2\frac{\Delta I}{I}-2\frac{\Delta \;\delta }{\delta }$$

If the sensor reports δ rather than the true $\delta^{\prime }$, the relative force bias to first order is

(15) $$\varepsilon_{F}\approx \frac{F\left(\delta^{\prime }\right)-F\left(\delta \right)}{F\left(\delta \right)}\approx 2\frac{\;\delta \;\prime -\delta \;}{\delta }$$

**Figure 5:**
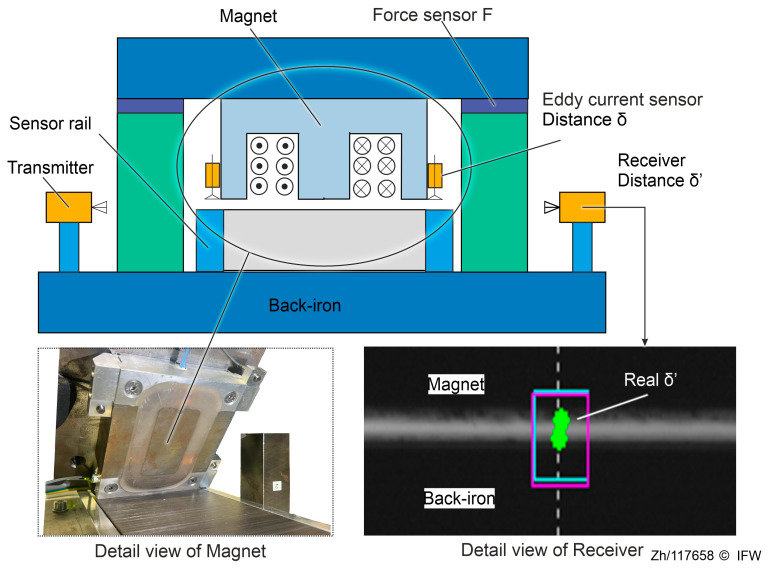
Test rig for a single magnet.

Therefore, even a gap contraction on the order of tens of micrometers can substantially alter force estimation and the linearization point (setpoint) in the high-force regime. In order to validate the assumptions of deformation and its influences, practical measurements were performed on a test rig using a single magnet and a small section of the guideway structure, as shown in Figure 5. In the first step, the extent of variation in the air gap, caused by the reluctance force, that can be detected using the eddy current sensors is examined. Later, the reliability of the measurements obtained with the eddy current sensors is evaluated by comparison with the optical shadow-casting measurement device.

The electromagnet is mounted on the top plate, with the eddy current sensors (Micro-Epsilon, Type ES08) attached to measure the distance between the magnet and the sensor rail. Three piezoelectric ring force sensors (Kistler, Type 9105A) are mounted on the top plate with fine thread screws attached to the electromagnet. The probe surfaces of the eddy current sensor are aligned with the magnet surface in the same horizontal plane. To prevent damage and displacement of the sensor due to collision, the measuring surface of the sensor rail is 0.1 mm lower than the surface of the back-iron.

On the bottom, the back-iron is mounted to the base plate using two T-bars, while on the base plate, two sensor rails are secured by bolts on either side. In addition to measuring the distance δ between the sensor rail and the eddy current sensor, an optical receiver and transmitter (Keyence, Type TM-040) are used to measure the real air gap δ’ directly. The magnet and back-iron structures are consistent with the actual structure used in magnetic guides; the technical specifications of the magnet are shown in Table 1.

**Figure 6:**
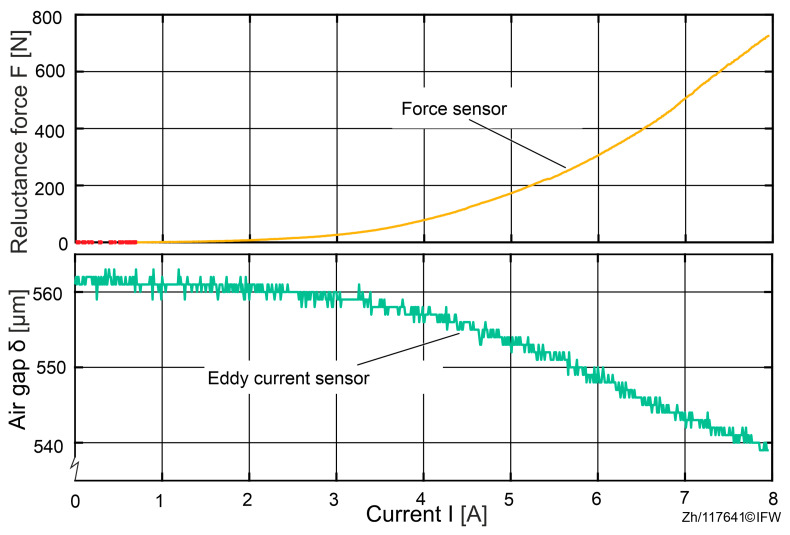
Air gap in relationship with increasing force.

In the initial state, the air gap between the magnet and the back-iron is measured as $\delta_{1}=561\;\mu m$ using three eddy current sensors. While the current of the magnet is increased from 0 to 8 A, both the air gap and the reluctance force are measured. The results are shown in Figure 6.

The results of the tests show that at a maximum current, a force of 727 N was achieved, while the average air gap drops to $\delta_{2}=540\;\mu m$. This suggests that under a reluctance force of 727 N, an air gap deviation of 21 μm between the sensor probe and the sensor rail can be detected. Since the observed variations in the air gap originate from the sensor probe and the sensor rail, and no direct measurements were taken between the magnet core and the back-iron, it can be assumed that the decrease in the air gap is attributable to the deformation of the test rig rather than to deformation of the magnet core or the back-iron.

Therefore, the impact caused by the deformation of the surrounding construction will be analyzed first. As the air gap narrows under increasing forces, the resulting magnetic reluctance force will exceed the theoretical expectation. With Maxwell’s formula for the iron-to-air transition tension and the actual data $\Delta \delta =\delta_{1}-\delta_{2}$, the theoretical reluctance force deviation $\Delta F_{Theoretical}$ due to deformation from test rig can be calculated:

(16) $$\Delta F_{Theoretical}=\frac{N^{2}\cdot A\cdot \mu_{0}}{8}\cdot \frac{I^{2}}{\delta_{1\;}^{2}}-\frac{N^{2}\cdot A\cdot \mu_{0}}{8}\cdot \frac{I^{2}}{\delta_{2\;}^{2}}\approx 39\;N$$

Based on the air-gap variations measured by the eddy current sensor, the deviation in the theoretical force estimates at 8 A can be computed. In the single-magnet rig of Section 4, the eddy current probe records changes in the probe-to-sensing-rail spacing and does not directly capture compression at the magnet core-to-back-iron interface, while the load cell simultaneously reports the actual magnetic force $F_{measured}$. Using the sensor-derived gap δ in Equation (1) yields the theoretical force $F_{estimation}\left(\delta \right)$. Comparison indicates that, at the maximum current of 8 A, the system achieves approximately 727 N of magnetic attraction. If only rig/fixture compliance (inferred from the eddy-current gap change) is considered, the theoretically explainable force increment is about 39 N. Therefore, under the influence of global test-rig deformation, the theoretically estimated reluctance force increases by approximately 39 N.

**Figure 7:**
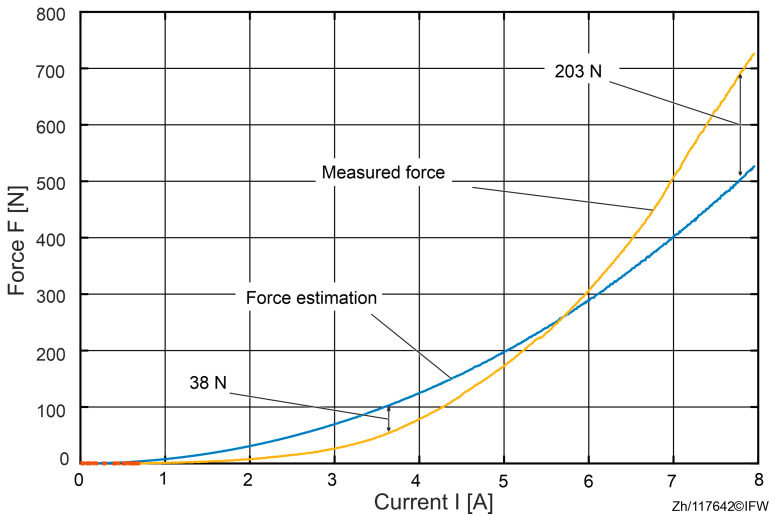
Comparison between actual and estimated reluctance force.

In order to analyze the force differences during an entire process from 0 A to 8 A, the actual and the theoretical reluctance force are compared in Figure 7. The theoretical reluctance force is based on Maxwell’s equations and the air gap measured with the eddy current sensor.

In the 0 to 8 A force–current sweep, the measured reluctance force rises more slowly than the simplified Maxwell-based estimate at the outset, with a maximum shortfall of about 38 N. This behavior is consistent with magnetic hysteresis: when the field is applied, material magnetization does not immediately follow external excitation but exhibits path dependence, yielding a delayed force buildup in the low current regime.

Once the current exceeds approximately 5.8 A, the measured force overtakes the Maxwell-based estimate and the discrepancy grows rapidly, reaching a peak deviation of 203 N at 8 A. This “5.8 A crossover” marks a transition from hysteresis-dominated small errors to deformation-dominated systematic bias, indicating that deformation-aware treatment should be incorporated into estimation and control in the mid-to-high current range.

At 8 A, a reproducible attribution partitions the 203 N deviation into three components: about 39 N (19.2%) arising from rig and fixture deformation (as inferred from eddy-current-based gap changes). Approximately 137 N (67.5%) is attributable to additional mechanical compliance within the magnetic assembly. Specifically, after subtracting the rig term, elastic deformation of the magnetic core and back-iron is required to reconcile the measurement with the Maxwell estimate. In addition, the residual of approximately 27 N (13.3%) is attributable to hysteresis and modeling simplifications.

The maximum force measured compared to the estimated discrepancy reaches 203 N, which is well beyond the 39 N attributable to test-rig compliance, implicating additional local compression within the magnetic assembly (core and back-iron) that makes the true gap smaller than the probe-reported gap. The magnitudes align with the trends in the plotted curves and are consistent, at the scaling level, with the established sensitivity relations.

Increasing excitation induces structural deformation that reduces the air gap. Because the eddy current probe does not capture elastic compression internal to the magnetic assembly, the Maxwell-based estimate underpredicts the actual reluctance force, thereby tightening clearances and elevating the collision risk. Key implication: the 39 N explained by rig deformation is far smaller than the total 203 N deviation; the dominant bias is unlikely to originate from the rig and more plausibly arises from elastic compression within the magnetic assembly (to be examined further in subsequent sections). This section reports observations and a first-order interpretation based solely on the eddy-current gap δ and load-cell force F. Direct measurement of the “true” gap and finer-grained quantification will be pursued in Section 5 using an independent optical shadow-casting system to validate and decompose the contributing effects.

## 5. Measurements and Solutions

In this section, the air gap deviation between the back-iron and the magnet is investigated. Due to the laminated construction and fastening methods of the back-iron, accurately analyzing its deformation through calculations or modelling is challenging. Furthermore, as mentioned in Section 3, capacitive or inductive sensors are unable to measure the deformation of ferromagnetic materials under complex electromagnetic conditions.

**Figure 8:**
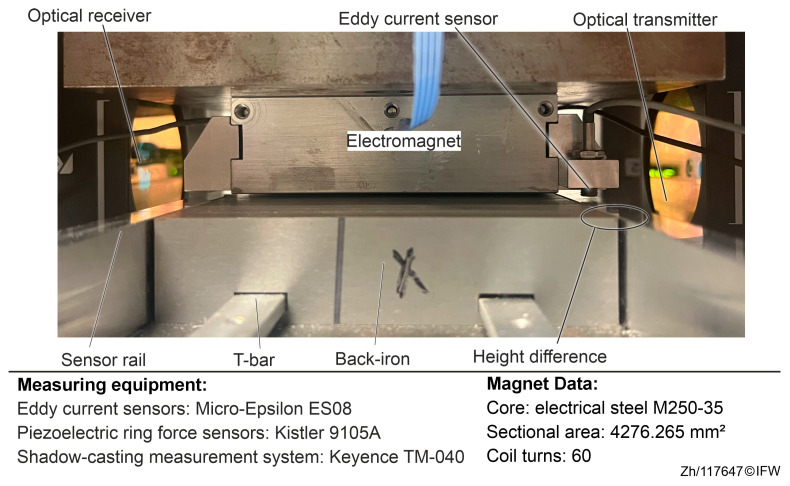
Shadow-casting measurement system.

Therefore, based on the construction of the magnetic guides, a shadow-casting measurement system (Keyence TM-040) with the optical transmitter and receiver and the setup in Figure 5 were used to analyze the deformation of the magnet core and the back-iron. The experimental setup is shown in Figure 8. The emitter and a receiver of the shadow-casting measurement system are positioned on either side of the single magnet. The shadow-casting measurement system measures the minimum distance between the magnet and the back-iron, representing the overall deformation at the air gap area. Additionally, the eddy current sensor next to the magnet measures the changes caused by the surrounding structural deformation but excludes the area of the magnet and the back-iron. The data measured by the eddy current sensor is used to isolate the effect of deformation in other areas from the variation in the air gap.

**Figure 9:**
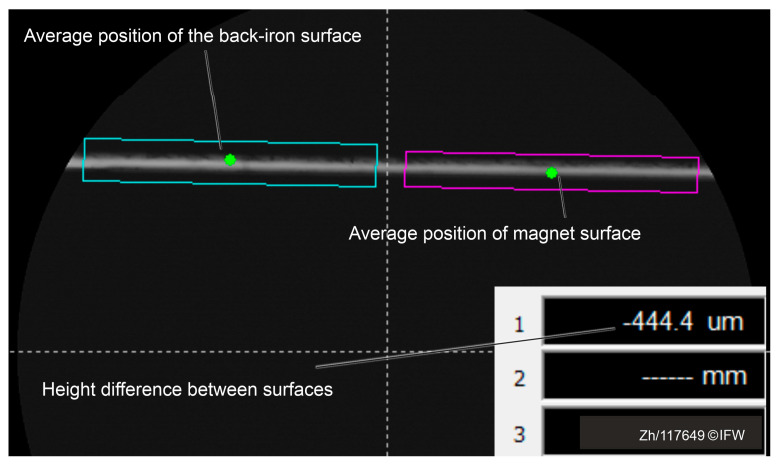
Average gap size on both sides of the gap.

In order to analyze the core and back-iron profiles, the eddy current sensor and the shadow-casting measurement system were calibrated. The optical path of the shadow-casting measurement system is adjusted parallel to the plane of magnet core. To minimize the influence of component and assembly tolerances, a wide measurement range was selected when setting the sampling points. Therefore, the average distance between the upper and lower surfaces can be obtained, as shown in Figure 9. The average heights of the upper and lower surfaces were taken from the left and right sides, respectively, to determine the height difference.

The back-iron surface is marked with a green dot in the blue search area, while the magnet core surface is marked with a green dot in the purple search area. The table in the bottom right corner of the image displays the height difference between the two surfaces. The first row shows a value of −444.4 µm, indicating that the back-iron surface is 444.4 µm lower than the magnet surface. The size of the air gap is obtained by the automatic recognition function of the measuring device.

**Figure 10:**
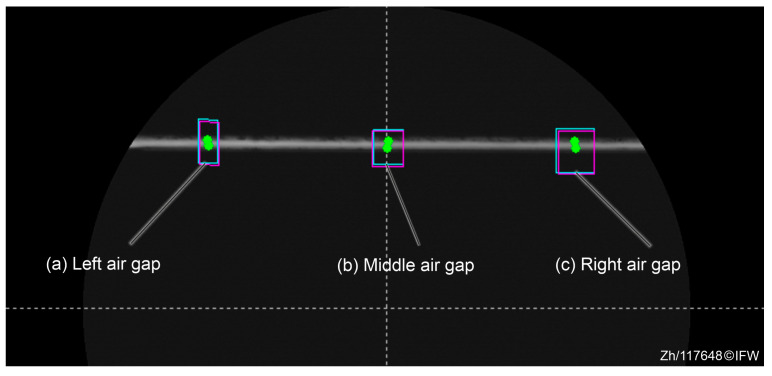
Measurement points.

The length of the air gap between the magnet core and the back-iron is divided into three sections (left, middle, right) to minimize the manufacturing and assembly tolerance. The air gap sizes in these regions are 440 μm, 449 μm, and 453 μm, respectively, in Figure 10. This maximum variation of 13 μm indicates potential errors in structural assembly or part processing. Therefore, in further analyses, the average value of all three heights was used to mitigate deviation errors.

The height of the support column was adjusted to match the previously tested position in Section 4 until both measurement systems recorded the value of the air gap at 561 µm. Thus, consistency in the measured data from both measurement systems when the magnet was not energized is ensured. Next, the coil current was continuously ramped from 0 A to 8 A. Measurements were taken at predefined sampling checkpoints spaced by 0.4 A. At each checkpoint the current was held for a dwell time to reach steady state, during which multiple images were recorded and averaged to reduce random signal fluctuations.

**Figure 11:**
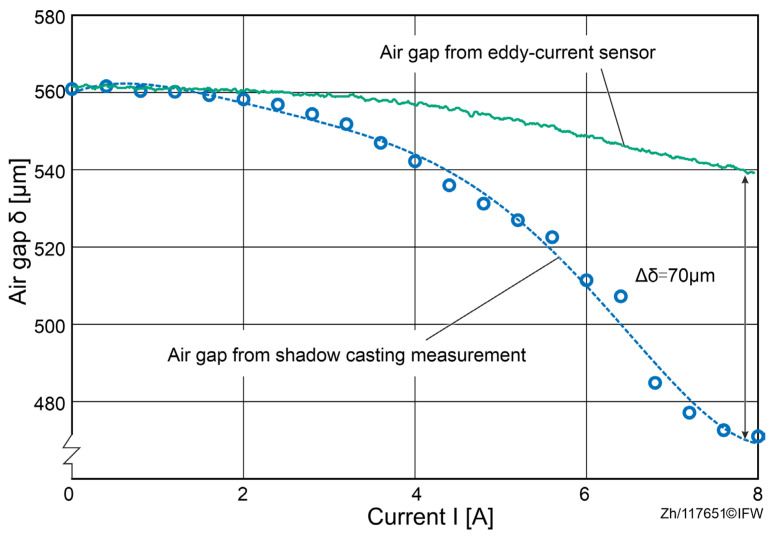
Comparison of the air gap from two different measurement systems.

The measured values from the shadow-casting measurement system gradually decrease with increasing current, from 561 μm at 0 A to 472 μm at 8 A, due to the corresponding deformation, as shown in Figure 11. For a current below 2 A, the deviation between the shadow-casting measurement system and the eddy current sensors remains under 2 μm, as the deformation of the back-iron and magnet core is negligible. However, when the current exceeds 2 A, the deviation increases up to 70 μm at 8 A. Considering the mounting position of the eddy current sensor and the laminated construction, this deviation can be attributed to the elastic deformation of the magnet core and the back-iron.

**Figure 12:**
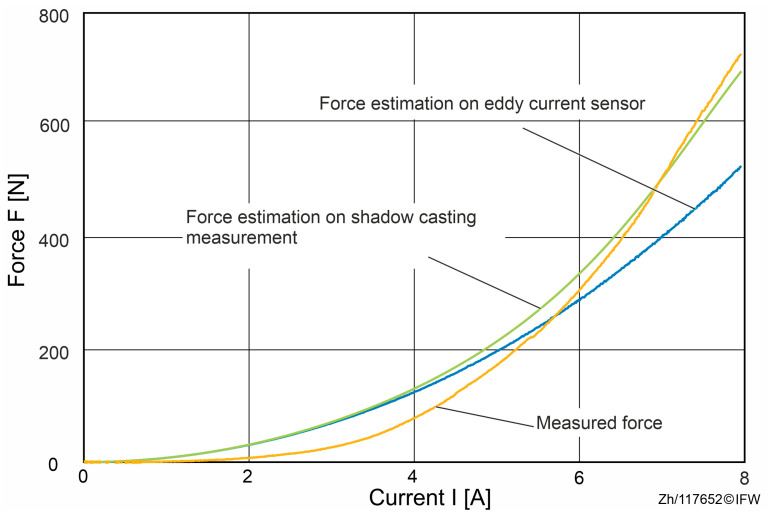
Force estimation based on eddy current sensor and shadow-casting measurements.

In addition, as shown in Figure 12, when the deformation of the magnet core and back-iron is considered and obtained with the shadow-casting measurement system, the estimated theoretical force is much closer to the measured reluctance force than the force estimated based on the data from the eddy current sensor.

Combining the previous assumptions and the two quantitative tests, it can be concluded that when using magnetic guides with a high reluctance force, the deformation of the magnet core and back-iron cannot be neglected. Otherwise, the air gap becomes smaller than the designed value, causing the actual generated reluctance force to increase. Additionally, it affects the maximum range of motion for magnetic guides. As the range of motion approaches its maximum value, contact between the magnet and the guideways can occur, resulting in friction and wear.

In applications with external process forces, the actual size of the air gap and accurate estimation of the reluctance force are key factors in ensuring the function of the magnetic guides. Any undetected state change will affect the actual set point position, thereby impacting the stability of levitation.

If the sensed gap is used in place of the true gap for linearization, the equivalent force–current gain is subject to systematic bias because the operating point is effectively interpreted as a “larger gap.” This manifests as an apparent gain increase, an upward shift in the setpoint, and an elevated risk of approaching the saturation boundary.

**Figure 13:**
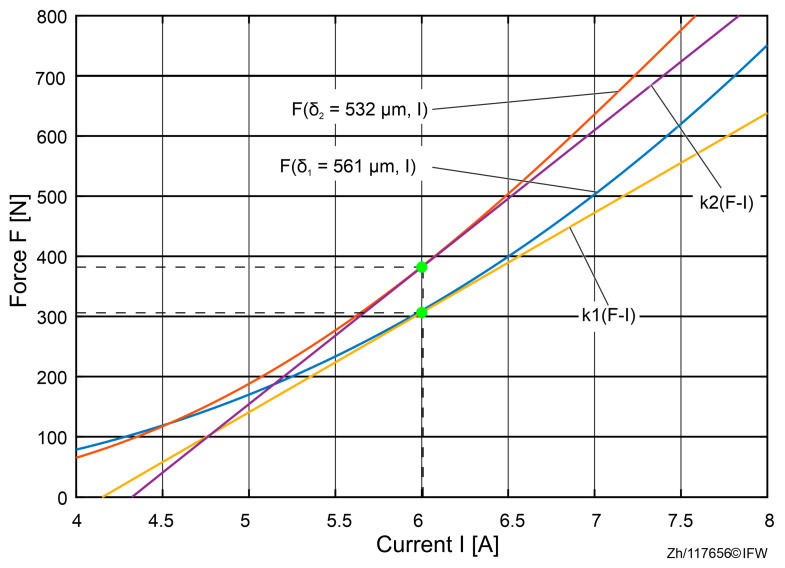
Shift in the set point.

In order to analyze the influence of air gap deviations on the set point, two cases with slightly different air gaps at δ= 561 μm  and δ=532 μm are chosen. The analyses in Figure 13 were conducted at 6 A. After the linearization of the corresponding set points, it can be seen that the force–current coefficient *k*_*i*(*F*-I)_ increases when the measured air gap is slightly smaller than the real value, which has an impact on the performance of the designed controller after linearization of the set point.

Therefore, in the high-load regime (≥5.8 A), conservative margins on the inner current loop and the outer PI/P controllers help suppress overshoot arising from linearization mismatch. The controller in magnetic guidance systems is usually a mature, modular unit with largely fixed internal tuning, which restricts direct retuning. Therefore, a residual-based supervisory strategy is preferred.

The force residual is defined as $r_{F}\left(I\right)=F_{estimation}\left(I\right)-F_{estimation}\left(\delta ,\;I\right)$. In the low-current range, $r_{F}$ remains negative with a small magnitude, consistent with hysteresis-dominated underperformance relative to the Maxwell-based estimate. As the current approaches approximately 5.8 A, $r_{F}$ crosses zero and then becomes positive, growing rapidly with excitation.

This zero-crossing identifies entry into a deformation-dominated region. Accordingly, control action can be partitioned by an operating zone: in the low-load segment where $|r_{F}|$ is small, no correction is applied and the existing tuning is retained; in the transition segment near 5.8 A, a conservative correction gain is introduced while monitoring the slope $r_{F}$. In the high-load segment, when $r_{F}>0$ and increases distinctly with current trenching, there is a maximum deviation of about 203 N near 8 A, which enables placeholder corrections to the current command and/or setpoint, while maintaining conservative margins in the inner and outer loops to mitigate deformation-induced bias.

The adaptive adjustment of the current based on the shift in the set point due to deformation should be conducted in applications. This will lead to the correct position of the set point and an accurate reluctance force estimation. Based on Equation (1), the magnitude of the corrected current $I_{2}$ can be obtained as:

(17) $$I_{2}=I_{1}\;\cdot \frac{\delta_{2}}{\delta_{1}}=\;I_{1}\;\cdot k_{\mathrm{correct}}\left(F,\delta \right)$$

where $I_{1}$ is the magnitude of the current in the ideal case and $\delta_{1}$ and $\delta_{2}$ are the magnitudes of the air gap before and after deformation.$\;k_{\mathrm{correct}}\left(F,\;\delta \right)$ is a correction function based on the reluctance force and the air gap.

**Figure 14:**
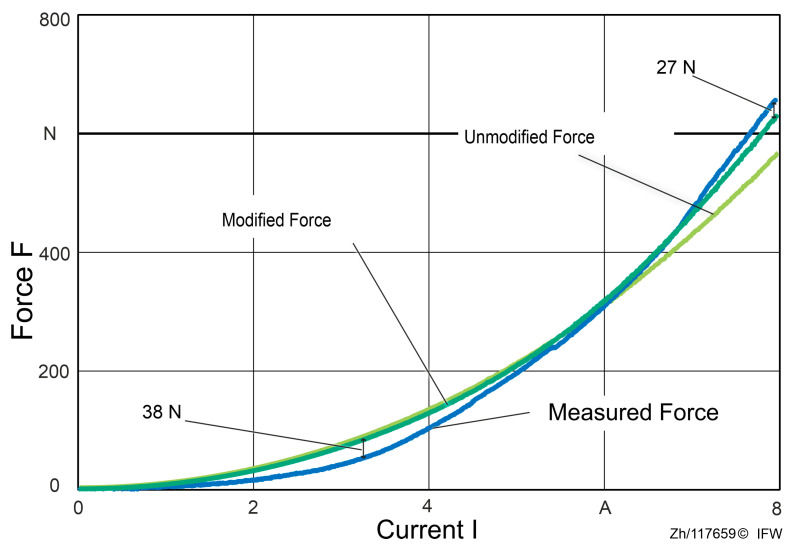
Modified force estimation considering the deformation of the magnet core and back-iron.

Considering the current correction method, repeated experiments were conducted under the same experimental conditions. After accounting for deformation and correcting the current accordingly, the estimated force showed better agreement with the measured force, as shown in Figure 14. In the basic working range (2 A to 8 A), the accuracy of the force estimation is improved. At 8 A, the maximal deviation is around 27 N. For small forces up to 200 N, the current requires little to no correction, and the estimation remains consistent with the previous results. However, when the applied force exceeds 200 N, the estimation method that considers the deformation model aligns closely with reality.

With this method, the force estimation error is minimized, particularly in regions where the reluctance force is high. Although a maximum deviation of up to 38 N still occurs, this discrepancy can be attributed to hysteresis effects and the idealized assumptions inherent in the analytical model. Therefore, when using an indirect measurement approach to determine the distance between the magnet and the back-iron in magnetic guide systems, the influence of structural deformations must also be considered to ensure accurate force estimation and reliable system performance.

## 6. Evaluation and Conclusions

This paper researches the impact of elastic deformation on magnetic guide systems, particularly focusing on the deformation of the magnet core and back-iron due to high reluctance forces. The experimental results confirm that laminated ferromagnetic materials, despite their advantages in reducing eddy current losses, are not ideal rigid bodies and exhibit significant deformation under high reluctance forces. During the experiment, a systematic divergence between the eddy current (EC) sensor and the optical measurement appears for forces above 200 N, evidencing an elastic decrease in the air gap due to structural compliance of the magnetic core, back-iron, and fixture.

When the EC-sensor value is used directly in the analytical force model under a rigid-body assumption, the estimated reluctance force overpredicts the measured force by approximately 39 N over a full current sweep (Figure 6, Figure 7 and Figure 12). For currents exceeding 5.8 A, a residual discrepancy of about 27 N (≈13.3%) remains, which can be attributed to magnetic hysteresis and modeling simplifications (e.g., the simplified Maxwell stress formulation). These results make clear that the decrease in the gap from elastic deformation is a dominant source of bias in conventional, EC-sensor-based force estimation.

In order to address this, a deformation-aware current adaptation was implemented that corrects the set-point drift caused by gap shrinkage and updates the control current accordingly. The adaptation effectively restores the intended linearization point and mitigates the variation in the effective gain k_i_. As shown in Figure 13 and Figure 14, the compensated estimation aligns much more closely with the measured force across the tested current range, demonstrating that explicitly accounting for elastic deformation substantially improves estimation accuracy and control consistency without hardware changes.

In conclusion, elastic deformation must be explicitly considered in the control of magnetic guides for high-precision machining, as neglecting it degrades force estimation, reduces collision margins, and can compromise stability. A software-only compensation was presented that combines set-point correction with current allocation to improve force consistency without hardware changes. The present validation used a single-axis (1D) reluctance-actuator rig under quasi-static excitation. Frequency-dependent eddy-current losses, fixture compliance, and lamination-bond variability were bound but not parameterized, and a simplified Maxwell stress formulation limited accuracy at high currents. Although the actuator is 1D, the workflow generalizes algorithmically to the coupled multi-DOF guidance case, which is consistent with the magnetic guidance’s role as a guiding and compensating subsystem. Through assembling per-gap measurements into a pose-dependent gap vector, set points will be updated via pose-dependent mapping to restore nominal clearances. Next, the currents through MIMO decoupling are allocated, accounting for geometric coupling and structural compliance, all without hardware redesign. Future work will refine real-time compensation using deformation feedback, advance optical sensing and sensor fusion for more accurate online gap estimation, and explore structural optimizations to mitigate deformation effects.

## Figures and Tables

**Table 1:** Technical specification of the active electromagnet.

Specification	Value	Unit
Maximum current I	8	[A]
Number of coils turns N	60	[/]
Cross-sectional area A	4276.265	[mm^2^]
Vacuum permeability μ_0_	4π × 10^−7^	[H/m]

## Data Availability

Data will be made available upon reasonable request to the corresponding author.
